# A case of PLA2R-positive membranous nephropathy with subsequent development of IgG4-related disease

**DOI:** 10.1007/s13730-024-00941-8

**Published:** 2024-11-08

**Authors:** Fumiaki Tanemoto, Imari Mimura, Hiroyuki Abe, Masaomi Nangaku

**Affiliations:** 1 Division of Nephrology and Endocrinology, The University of Tokyo Hospital, 7-3-1 Hongo, Bunkyo-Ku, Tokyo, 113-8655 Japan; 2 Division of Pathology, The University of Tokyo Hospital, 7-3-1 Hongo, Bunkyo-Ku, Tokyo, 113-8655 Japan

**Keywords:** Membranous nephropathy, Nephrotic syndrome, IgG4-related disease, Autoimmune pancreatitis, Phospholipase A2 receptor (PLA2R), Primary membranous nephropathy

## Abstract

Membranous nephropathy (MN) is a common cause of adult-onset nephrotic syndrome. It is also known as a minor but established renal manifestation of Immunoglobulin G4-related disease (IgG4-RD). Previous reports suggest that MN can also be an initial manifestation of IgG4-RD, all of which are phospholipase A2 receptor (PLA2R)-negative MN. We describe a case of PLA2R-positive MN that subsequently developed other manifestations of IgG4-RD. A 60-year-old male with nephrotic syndrome was diagnosed as primary MN with positive staining for PLA2R on the initial renal biopsy, which remained in partial remission with supportive therapy using angiotensin II receptor blocker (ARB) without steroid. About 1 year later, a renal mass was detected during an annual checkup, and contrast-enhanced computed tomography revealed low-density masses in bilateral kidneys and the head of the pancreas. The findings of endoscopic biopsy of the pancreatic mass were consistent with autoimmune pancreatitis (AIP) and the second renal biopsy showed the findings of MN with tubulointerstitial nephritis, both of which led to a diagnosis of IgG4-RD. The second renal biopsy also showed positive PLA2R. The patient received oral glucocorticoid therapy for IgG4-RD, which improved IgG4-related AIP and renal masses and also resulted in complete remission of MN. To our knowledge, this is the first reported case of PLA2R-positive MN with subsequent development of IgG4-RD. It is sometimes difficult to determine whether PLA2R-positive MN occurring with IgG4-RD is primary MN or secondary MN associated with IgG4-RD. The possibility of developing IgG4-RD should be considered even when preceding MN is PLA2R-positive, suggesting of primary MN.

## Introduction

Immunoglobulin G4-related disease (IgG4-RD) is an increasingly recognized systemic immune-mediated disorder characterized by dense infiltration of IgG4-secreting plasma cells into multiple organs [1, 2]. The affected organs often develop mass-like lesions with characteristic “storiform” fibrosis on histologic examination [3]. Various organs throughout the body can be affected, including the pancreas, lacrimal glands, salivary glands, biliary tract, lung, and kidney [4]. Early recognition and therapeutic intervention are essential for IgG4-RD because it is a treatable disorder; many of the clinical manifestations of IgG4-RD respond well to glucocorticoids, especially in the early stages of disease [2]. However, IgG4-RD is often difficult to detect due to its indolent nature and poorly understood pathogenesis, resulting in underdiagnosis [3].

Renal involvement is considered as one of the key findings of IgG4-RD; IgG4-related kidney disease (IgG4-RKD) includes tubulointerstitial nephritis (TIN), membranous nephropathy (MN), renal masses, and retroperitoneal fibrosis [3]. In particular, TIN with infiltration of IgG4-positive plasma cells is the most frequent renal manifestation. On the other hand, MN is a much less common but established manifestation of IgG4-RKD. IgG4-related MN sometimes coexists with IgG4-related TIN; according to a review of 34 previously reported cases of IgG4-related MN, 62% were coexistent with TIN and 38% were MN alone without TIN [6].

MN is the most frequent cause of nephrotic syndrome in adults and characterized by accumulation of subepithelial immune complexes composed of IgG and a target antigen along the glomerular basement membrane (GBM) [7]. About 80% of MN cases are primary MN, which is regarded as a kidney-specific autoimmune disease that mediated by autoantibodies to podocyte antigens; the most common target antigen is the M-type phospholipase A2 receptor (PLA2R), accounting for 85% of cases [8]. The remaining 20% of MN cases are secondary MN associated with other diseases, such as systemic infections, malignancies, and autoimmune disease, or exposures to drugs or toxins [8]. Glomerular staining for PLA2R is useful for distinguishing between primary and secondary MN, and PLA2R positivity strongly suggests the possibility of primary MN [9]. In fact, most cases of MN associated with IgG4-RD reported to date are PLA2R negative, which is similar to the case of other secondary MN due to other causes [5, 6].

Here, we report a quite rare case in which PLA2R-positive MN antedated autoimmune pancreatitis (AIP) and renal masses due to IgG4-RD. The potential relationship between PLA2R-positive MN and IgG4-RD is discussed.

## Case report

**Fig. 1:**
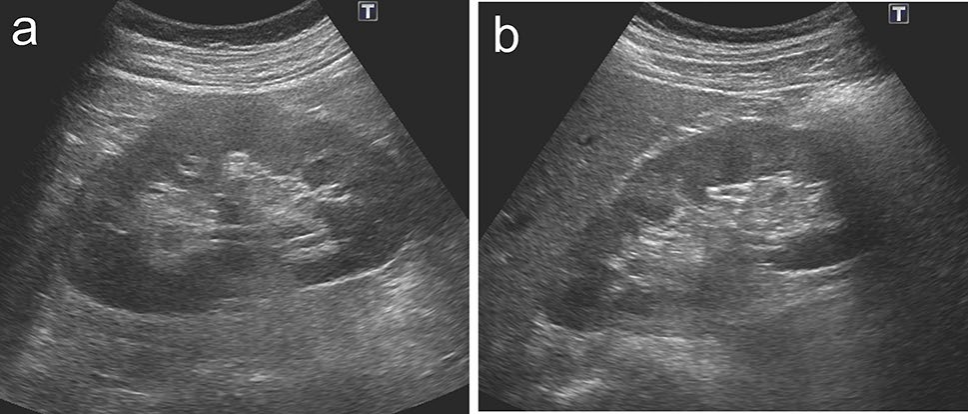
Abdominal ultrasound findings at the time of the initial renal biopsy. **a**, **b** No mass lesions were observed in the left kidney (**a**) and the right kidney (**b**)

**Fig. 2:**
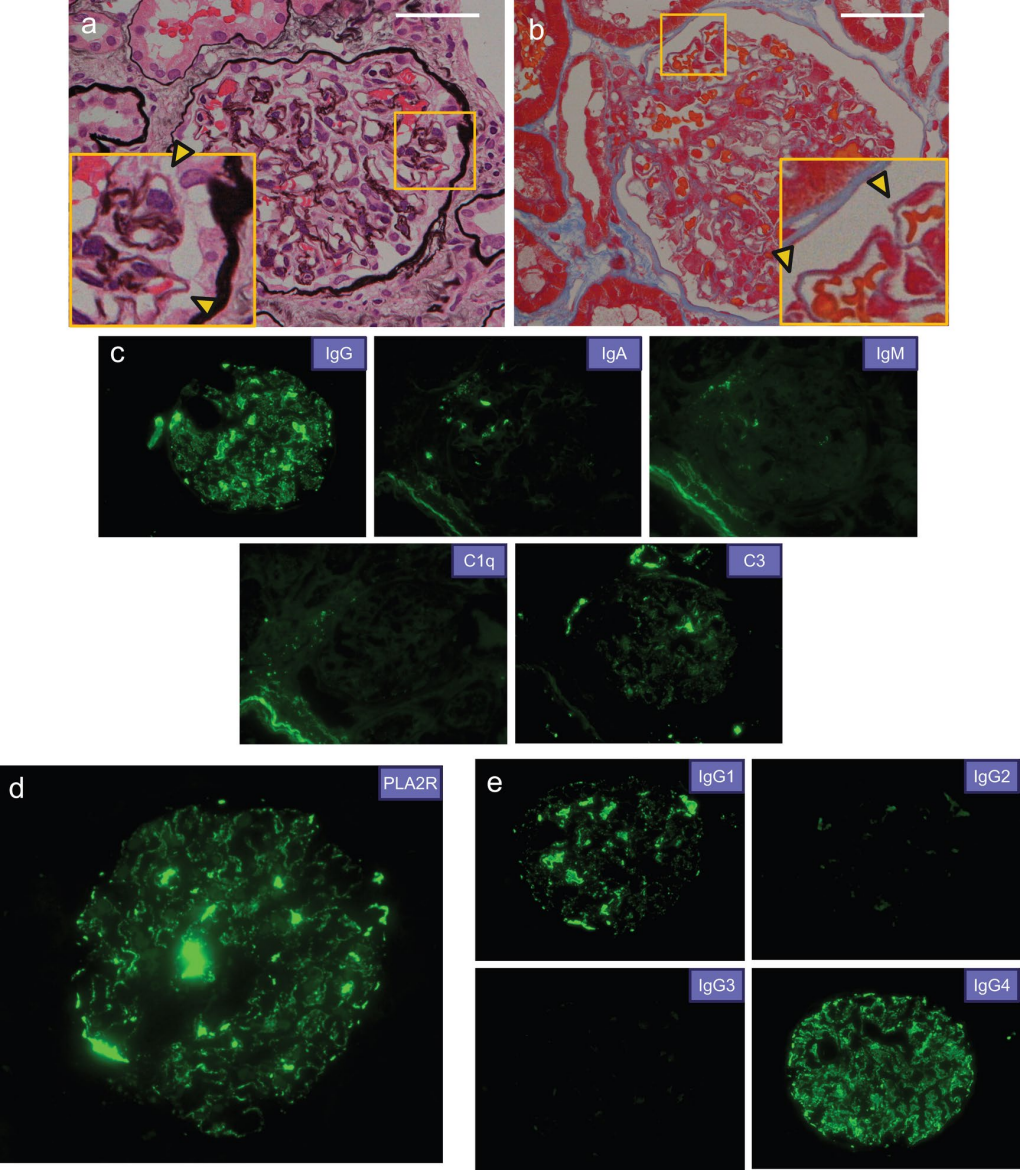
Histopathologic findings of the initial kidney biopsy at the onset of MN. **a** Scattered fluffing (yellow arrow) on the capillary walls was observed. The area enclosed by yellow square is shown enlarged. Periodic acid-methenamine-Masson trichrome (PAM) stain, × 400, scale bar 50 µm. **b** Granular deposits (yellow arrow) were detected. The area enclosed by yellow square is shown enlarged. Azan stain, × 400, scale bar 50 µm. **c** Immunofluorescence (IF) staining showed IgG(+), IgA(−), IgM(−), C1q(−), and C3(+) in the capillary walls. **d** IF staining showed PLA2R(+) in the capillary walls. **e** IF staining for IgG subclasses showed predominant positive staining for IgG4, followed by IgG1. C1q, complement C1q; C3, complement C3; IgA, immunoglobulin A; IgG, immunoglobulin G; IgM, immunoglobulin M; PLA2R

In June 2021, a 60-year-old man with no relevant past medical history was referred to our department because of proteinuria detected during a health check. He had been aware of pitting edema of the lower extremities and frothy urine for about a month prior to the visit. It was the first time that an abnormal urinalysis had been detected during his annual health check. Laboratory investigations showed serum albumin of 2.5 g/dL, creatinine of 0.65 mg/dL, LDL cholesterol of 216 mg/dL. Urine tests showed urinary protein creatinine ratio (UPCR) of 5.0 g/gCr and no hematuria. An abdominal ultrasound performed at this time showed no mass lesions in the kidneys (Fig. 1). Percutaneous kidney biopsy was performed to investigate the cause of his nephrotic syndrome. Light microscopy showed scattered fluffing and particle-like deposition on the capillary walls (Fig. 2a, b). The tubulointerstitium showed no significant fibrosis or lymphocyte infiltration. Immunofluorescence (IF) staining showed diffuse granular staining of IgG (IgG1(1+), IgG2(−), IgG3(−), IgG4(2+)) and C3 along the glomerular capillary walls, and they were positive for PLA2R (Fig. 2c–e). Electron microscopy (EM) findings were not available because no glomeruli were included in the sample submitted for EM. The pathological diagnosis was MN, and PLA2R positivity and IgG4-predominant deposition strongly supported the diagnosis of primary MN.

**Fig. 3:**
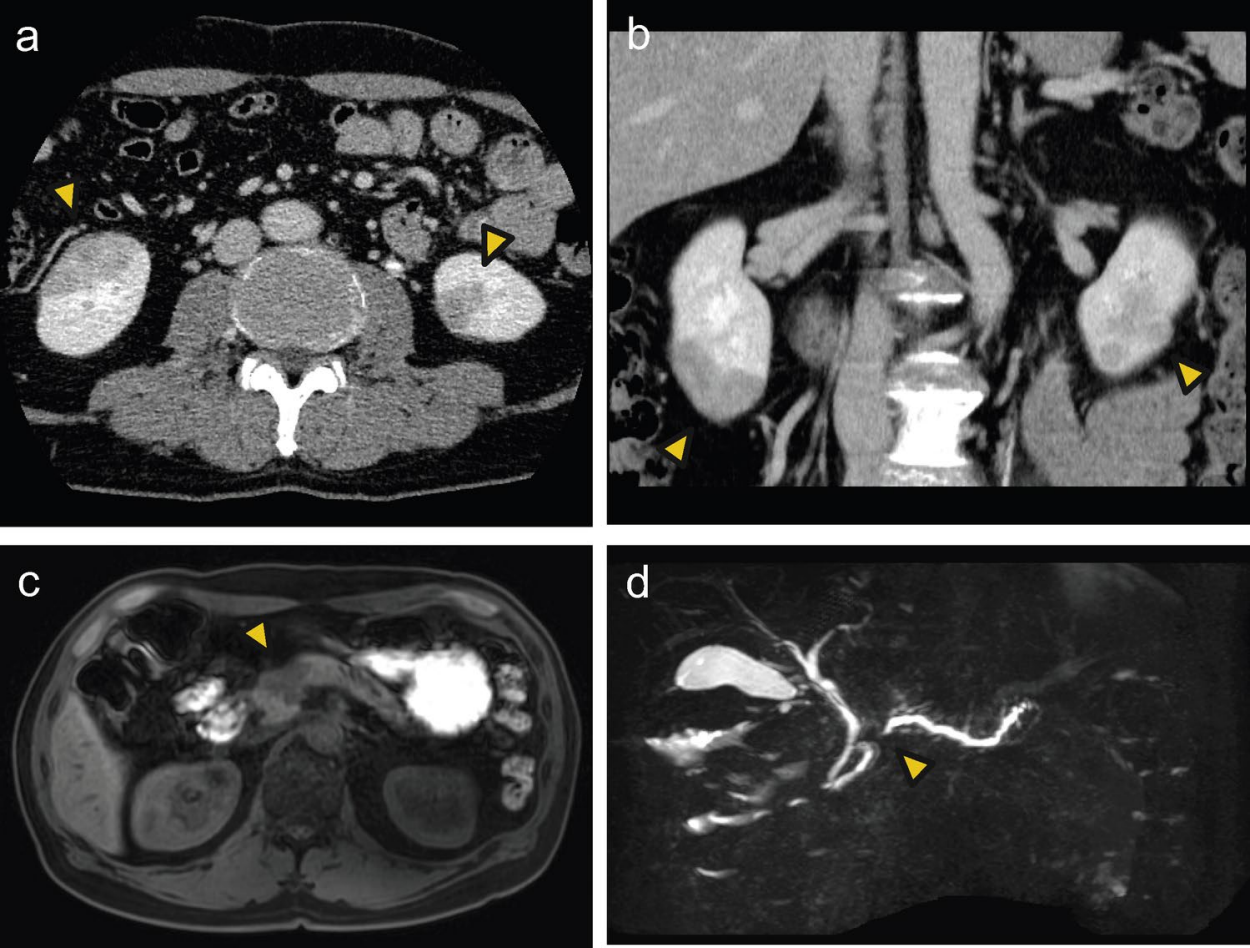
Contrast-enhanced CT and MRI of the abdomen highlighting masses in the kidney and the pancreas. **a**, **b** Bilateral renal hypovascular masses (yellow arrow) were detected on a contrast-enhanced CT. **c**, **d** Pancreatic hypovascular mass in the head of the pancreas and a narrowing of the main pancreatic duct at the head of the pancreas were detected on MRI

The patient started taking losartan as a supportive therapy for the diagnosis of primary MN. Steroid treatment was not initiated at this time due to concerns about exacerbation of asymptomatic esophageal candidiasis discovered during upper gastrointestinal endoscopy. The supportive therapy resulted in a decrease in proteinuria and partial remission of nephrotic syndrome, but MN did not resolve spontaneously and proteinuria of about 1‒2 g/gCr continued. In June 2022, a hyperechoic mass was detected in the right kidney during an abdominal ultrasound during health checkup. A contrast-enhanced CT performed in December 2022 revealed a mass with irregular margin and poor contrast in the right kidney and a similar mass in the left kidney (Fig. 3a, b). It also detected poorly enhanced mass in the head of the pancreas with dilated main pancreatic duct; a magnetic resonance imaging (MRI) of the abdomen revealed a mass with a significant narrowing of the main pancreatic duct at the head of the pancreas (Fig. 3c, d).

Endoscopic ultrasound-guided fine-needle aspiration (EUS-FNA) was performed to examine the detected pancreatic mass. The specimen of the initial biopsy was small and insufficient for a histological diagnosis, but cytological examination revealed class I and no evidence of malignancy. In February 2023, the patient underwent a second EUS-FNA. The second biopsy findings were marked by acinar atrophy, interstitial fibrosis, and extensive lymphoplasmacytic infiltration with IgG4-positive plasma cells. Localized swelling of the pancreas, irregular stenosis of the main pancreatic duct, elevated serum IgG4 levels (187 mg/dL), and histological findings with prominent infiltration of lymphocytes and IgG4-positive plasma cells (> 10 cells per high-power field [HPF]) led to a definite diagnosis of IgG4-RD and IgG4-related AIP, based on the 2020 revised comprehensive diagnostic (RCD) criteria for IgG4-RD and organ-specific criteria (Japanese clinical diagnostic criteria for autoimmune pancreatitis, 2018) [10, 11].

**Fig. 4:**
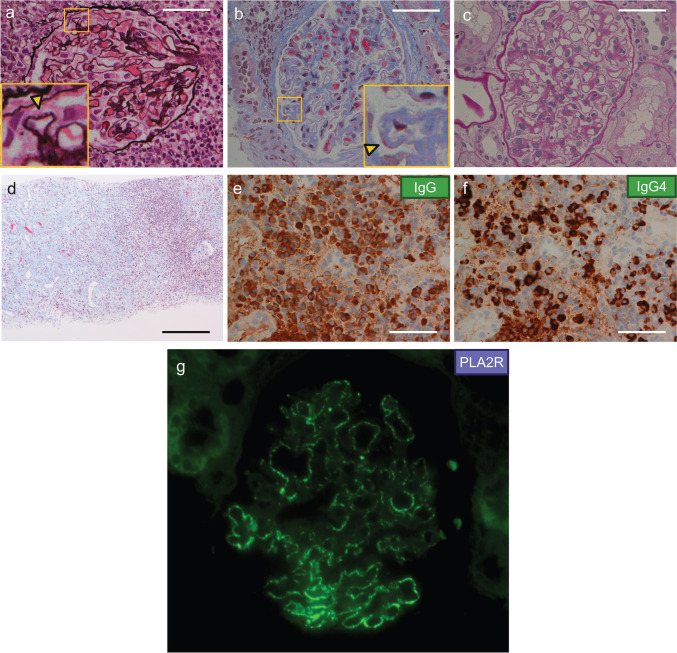
Histopathologic findings of the second kidney biopsy after the onset of IgG4-related AIP and renal masses. **a** The thickening of GBM was noted. Spike formation (yellow arrow), fluffing, and particle-like deposition were observed on the capillary walls. The area enclosed by yellow square is shown enlarged. PAM stain, × 400, scale bar 50 µm. **b** Granular deposits (yellow arrow) were observed on the capillary walls. The area enclosed by yellow square is shown enlarged. Masson’s trichrome stain, × 400, scale bar 50 µm. **c** No significant proliferation of mesangial cells or matrix were observed in glomeruli. PAS stain, × 400, scale bar 50 µm. **d** The tubulointerstitium was marked by a dense lymphoplasmacytic cell infiltration. Masson’s trichrome staining, × 100, scale bar 200 µm. **e**, **f** Immunohistochemical staining for IgG and IgG4 revealed increased number of IgG4-positive plasma cells of about 100 cells/HPF and an IgG4/IgG ratio of about 50% in the interstitium. × 400, scale bar 50 µm. **g** Immunofluorescence staining showed PLA2R(+) in the capillary walls. IgG, immunoglobulin G

In March 2023, a CT-guided biopsy of renal mass in the left kidney was performed. Cytological examination showed class I and no evidence of malignancy. This second renal biopsy sample showed thickening of glomerular capillary walls with spikes, fluffing, and particle-like deposition (Fig. 4a, b). There was no significant proliferation of mesangial cells or matrix (Fig. 4c). These findings were consistent with MN, and the membranous lesions were more distinct than those of the initial biopsy. Moreover, the tubulointerstitium was marked by a dense lymphoplasmacytic cell infiltration, including IgG4-positive plasma cells, accompanied by tubular atrophy (Fig. 4d–f). IF staining of PLA2R was positive in the glomerular capillary wall, similar to the initial renal biopsy (Fig. 4g). The presence of renal hypovascular masses, elevated serum IgG4 level, histological findings of TIN with dense infiltration of lymphocytes and IgG4-positive plasma cells (> 10 cells per HPF), and extra-renal organ findings compatible with AIP led to a definite diagnosis of IgG4-RKD, based on the diagnostic criteria for IgG4-related kidney disease (IgG4-RKD) 2020 [12].

**Fig. 5:**
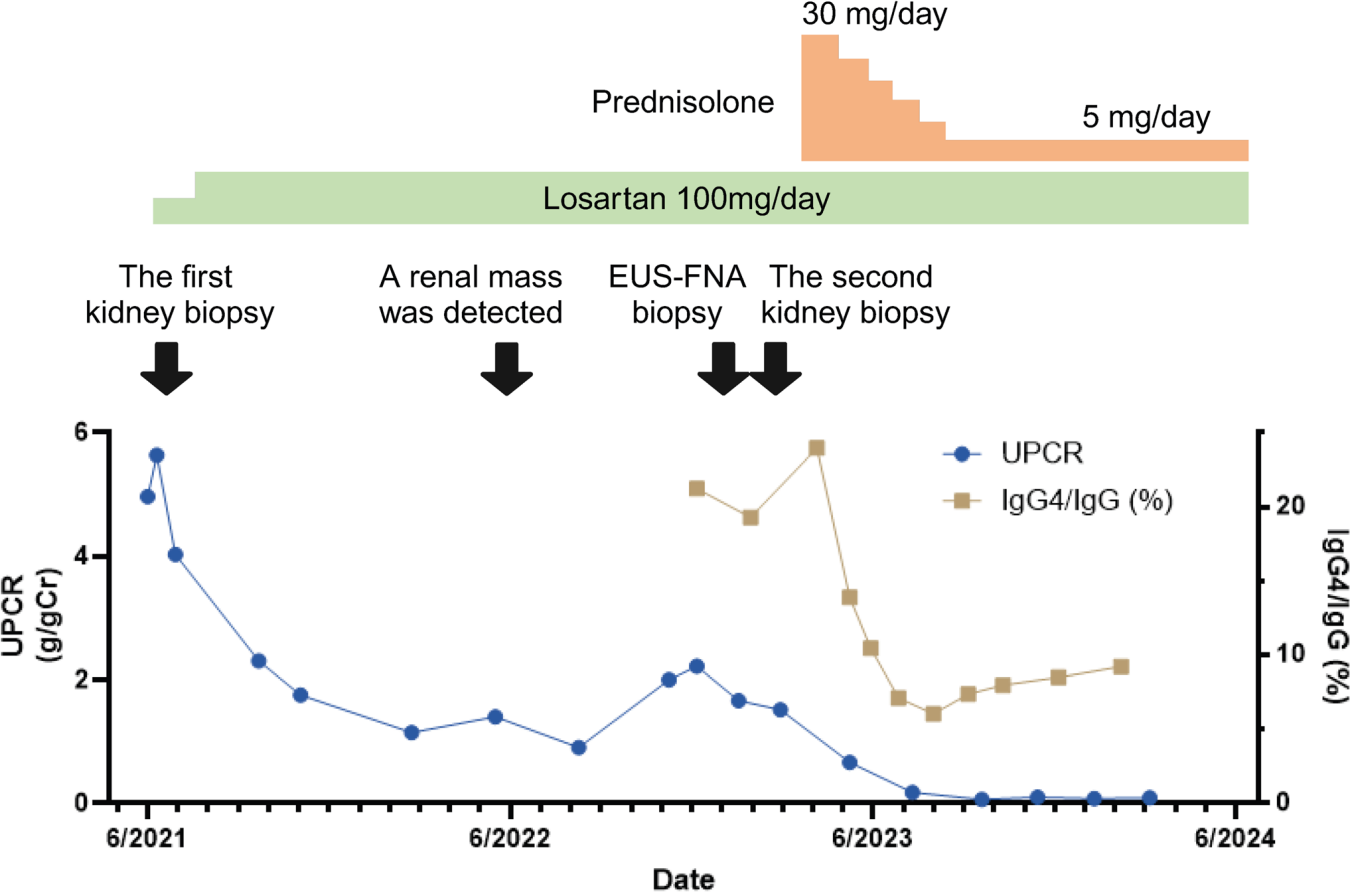
Changes in the amount of proteinuria (UPCR) and serum IgG4 level over time. Losartan alone did not induce remission of MN, but glucocorticoid therapy was initiated and proteinuria and IgG4 levels rapidly improved. EUS-FNA, endoscopic ultrasound-guided fine-needle aspiration; IgG, immunoglobulin G; UPCR, urinary protein creatinine ratio

Treatment with prednisolone 30 mg (0.5 mg/kg) daily was started for IgG4-related AIP and renal masses in April 2023, when the patient’s laboratory data showed serum creatinine of 0.71 mg/dL, IgG of 1215 mg/dL, IgG4 of 291 mg/dL, IgG4/IgG ratio of 24.0%, and UPCR of 1.5 g/gCr on losartan (Fig. 5). One month after the start of treatment, CT and magnetic resonance cholangiopancreatography (MRCP) confirmed improvement in the size of renal masses and pancreatic mass and irregularity in the caliber of the main pancreatic duct. IgG4 decreased to 66 mg/dL at that time. Proteinuria also improved after starting prednisolone and reached complete remission 3 months later, suggesting that glucocorticoid therapy for IgG4-RD also improved MN. The patient has been achieved complete remission of MN for about 1 year on a maintenance dose of prednisolone 5 mg daily. The clinical course is summarized in Table 1.

**Table 1:** Clinical course and laboratory findings of the present case

		2020/7	2021/6	2022/6	2022/12	2023/3	2023/4	2023/7	2024/3
Event		Annual health check	The initial kidney biopsy: MN	Detection of renal mass	Detection of pancreatic mass	The second kidney biopsy: MN + TIN	Before starting prednisone for IgG4-RD	3 months after starting prednisone	1 year after starting prednisone
TP (g/L)	(NR: 6.6–8.1)	6.8	5.1	6.1	6.4	5.8	6.2	5.8	7.1
Alb (g/L)	(NR: 4.1–5.1)	4.0	2.5	3.3	3.4	2.8	3.2	3.6	4.4
LDL-C (mg/dL)	(NR: 65–139)	84	216	132	179	172	131	154	96
Cr (mg/dL)	(NR: 0.65–1.07)	0.68	0.65	0.62	0.63	0.69	0.71	0.75	0.69
eGFR (mL/min/1.73 m^2^)		91.8	96	100.6	98.8	89.1	86.3	81.3	88.7
IgG (mg/dL)	(NR: 861–1747)	ND	948	ND	1174	970	1215	648	1041
IgG4 (mg/dL)	(NR: 11–121)	ND	ND	ND	249	187	291	46	96
IgG4/IgG (%)		ND	ND	ND	21.2	19.3	24.0	7.1	9.2
IgE (mg/dL)	(NR: 0–170)	ND	ND	ND	505	ND	418	ND	ND
C3 (mg/dL)	(NR: 73–138)	ND	140	ND	130	ND	143	ND	ND
C4 (mg/dL)	(NR: 11–31)	ND	28	ND	26	ND	27	ND	ND
Urinalysis (protein)		(−)	(2+)	(2+)	(2+)	(2+)	ND	(1+)	(−)
UPCR (g/gCr)		ND	4.97	1.40	2.22	1.51	ND	0.17	0.08
Treatment		None	None	Losartan	Losartan	Losartan	Losartan	PSL, Losartan	PSL, Losartan

Alb, albumin; Cr, creatinine; eGFR, estimated glomerular filtration ratio; LDL-C, low-density lipoprotein cholesterol; PSL, prednisolone; NR, normal range; TP, total protein

## Discussion

To our knowledge, this is the first reported case of PLA2R-positive MN which subsequently developed IgG4-RD. It is quite difficult to distinguish whether this patient’s renal disease is primary MN or secondary MN related to IgG4-RD. It may simply be primary MN complicated later by IgG4-RD, which might suggest the presence of a common pathology between primary MN and IgG4-RD. Alternatively, it may be renal involvement that precedes other manifestations of IgG4-RD. In this regard, this case showed an atypical course for IgG4-related secondary MN in the following two respects: 1) glomerular staining for PLA2R was positive; and 2) MN alone preceded other manifestations of IgG4-RD.

At the onset of nephrotic syndrome, both the clinical course without extrarenal diseases and renal biopsy findings of PLA2R-positive MN without TIN strongly suggested a diagnosis of primary MN [8, 13]. Predominant deposition of IgG4 followed by IgG1 in IgG subclass staining was consistent with both primary MN and IgG4-related secondary MN [14]. The clinical course after the initial renal biopsy that showed partial remission of nephrotic syndrome with losartan may be explained by the effect of ARB, but it could be due to spontaneous resolution of primary MN.

Positive glomerular PLA2R staining is highly specific for the diagnosis of primary MN [8, 9], and actually, most cases of MN in the setting of IgG4-RD have been reported to be negative for PLA2R [6]. However, five cases have been reported to date in which PLA2R-positive MN developed during the course of IgG4-RD (Table 2) [14–18]. Although some of these cases may actually be PLA2R-associated primary MN unrelated to IgG4-RD, some features of these cases are uncommon for primary MN. For example, the presence of subendothelial and mesangial deposits on EM, observed in Case 5, is atypical for primary MN (Table 2) [9, 18]. Moreover, the favorable response of MN to steroid treatment for IgG4-RD observed in Case 5 and our case suggests that these are more likely to be IgG4-related secondary MN rather than primary MN. In fact, a certain number of PLA2R-positive cases are reported in secondary MN due to other diseases, including hepatitis B, hepatitis C, and sarcoidosis, possibly via inducing immune response to PLA2R [19]. For example, in sarcoidosis, a related disease of IgG4-RD, PLA2R-positive MN is frequently observed in active sarcoidosis, which suggests the existence of a causal relationship in which the immunological status of sarcoidosis may induce immunization against PLA2R [20]. As is the case with secondary MN associated with sarcoidosis, the possibility cannot be ruled out that there is PLA2R-positive secondary MN associated with IgG4-RD. Positive PLA2R on the second renal biopsy after the onset of IgG4-RD may suggest the possibility of PLA2R-positive secondary MN related to IgG4-RD.

**Table 2:** Summary of previously reported cases of PLA2R-positive MN associated with IgG4-RD

Case no	Age/gender	sCr at diagnosis of MN (mg/dL)	Serum IgG/IgG4 at diagnosis of MN (mg/dL)	Proteinuria at diagnosis of MN	Order of onset of TgG4-RD and MN	Tissue PLA2R	Serum PLA2R	TIN	IgG subclasses by IF	Distribution of EDDs in EM	Treatment	Time to achieve remission	Ref
1	64, M	2.58	435/25	12 g/24 h	IgG4-RD → MN (4 years)	ND	(+)	(+)	**1(+)** predominance	ND	PSL (1 mg/kg) + CY	6 months	[15]
2	45, M	0.9	551/28.9 (onset of IgG4-RD)	7 g/gCr	IgG4-RD → MN (5 months)	(+)	(+)	(−)	1(−), 2(−), 3(−), **4(2+)**	endo, mes (−)	PSL + RTx + CY	1 year	[14]
3	69, M	1.24	1760/170	12.8 g/24 h	IgG4-RD = MN	(+)	ND	(+)	**1(2+)**, 2( ±), 3(+), **4(2+)**	endo, mes (−)	MP + PSL + LDL-A	Not achieved (3.44 g/gCr)	[16]
4	55, M	Within normal range	ND/836.9	6.4 g/gCr	IgG4-RD = MN	(+)	(−)	(+)	4(+), others: ND	ND	PSL + CNI + RTx + Obi	2 years 6 months	[17]
5	77, M	1.67	1154/451.3	10.5 g/gCr	IgG4-RD = MN	(+)	(+)	(−)	**1(2+)**, 2(+), **3(2+)**, **4(2+)**	endo, mes (+)	PSL (0.5 mg/kg)	Within 1 year	[18]
6	60, M	0.65	1st: 948/ND 2nd: 970/187	5.0 g/gCr	MN → IgG4-RD (1 year)	(+)	ND	1st: (−) 2nd: (+)	1(+), 2(−), 3(−), **4(2+)**	ND	PSL (0.5 mg/kg)	3 months	This case

CNI, calcineurin inhibitor; CY, cyclophosphamide; EDDs, electron dense deposits; endo, subendothelial deposits; LDL-A, low-density lipoprotein apheresis; mes, mesangial deposits; ND, not described; Obi, obinutuzumab; PSL, prednisolone; RTx, rituximab; sCr, serum creatinine; 1st, the initial renal biopsy; 2nd, the second renal biopsy

Furthermore, this case of PLA2R positivity on both the initial and second renal biopsy suggests that renal damage at either time point has a common etiology. Regarding the onset of IgG4-related secondary MN, there have been several reports of rare cases in which the onset of MN precedes the onset of other symptoms of IgG4-RD, suggesting that MN can be an initial manifestation of IgG4-RD (Table 3) [21–23]. All three cases reported so far are predominantly positive for IgG4 by tissue staining and negative for PLA2R by serum or tissue staining, consistent with the characteristics of IgG4-related secondary MN. In these cases, time interval from the onset of MN to the onset of IgG4-RD ranges from 1 to 3 years, which is similar in this case. The clinical course of this case, in which the second biopsy showed worsening MN and new development of TIN compared with the first one, has been observed in Case 3 (Table 3). These three cases suggest that IgG4-RD may contribute to the pathogenesis of MN through some mechanism, even at a subclinical status.

**Table 3:** Summary of previously reported cases of MN preceding the onset of other manifestations of IgG4-RD

Case no	Age/gender		Time interval from MN to IgG4-RD	sCr (mg/dL)	Serum IgG/IgG4 (mg/dL)	Proteinuria	Tissue PLA2R	Serum PLA2R	TIN	IgG subclasses by IF	Distribution of EDDs in EM	Treatment	Time to achieve remission	Ref
1	59, M	Onset of MN	3 years	CCr 90.5 mL/min	1198/ND	4.3 g/day	ND	ND	(−)	1(+), 2(−), 3(−), 4(+)	endo, mes (−)	PSL (40 mg)	Not achieved (3 g/day)	[21]
		Onset of IgG4-RD		1.13	3921/1920	2.1 g/day	ND	(−)	ND	ND	ND	PSL (40 mg)	Not achieved (3 g/day)	
2	70, M	Onset of MN	3 years	ND	ND/186	ND	(−)	(−)	(−)	4(+) by IHC	ND	MP + CY	Achieved	[22]
		Onset of IgG4-RD		ND	ND/1220	ND	ND	ND	ND	ND	ND	PSL (40 mg)	ND	
3	46, M	Onset of MN	1 year 5 months	1.09	500/ND	5.2 g/day	ND	(−)	(−)	ND	mes (+)	PSL (60 mg) + CY	11 months	[23]
		Onset of IgG4-RD		3.85	1620/25.5	11.8 g/day	ND	(−)	(+)	4(+)	endo, mes (+)	PSL + CY	5 months	
4	60, M	Onset of MN	1 year	0.65	948/ND	5.0 g/gCr	(+)	ND	(−)	1(+), 2(−), 3(−), 4(2+)	ND	Losartan	Not achieved (1 g/gCr)	This case
		Onset of IgG4-RD		0.69	970/187	1.5 g/gCr	(+)	ND	(+)	4(+) by IHC	ND	PSL (0.5 mg/kg)	3 months	

CCr, creatinine clearance; CY, cyclophosphamide; EDDs, electron dense deposits; endo, subendothelial deposits; IHC, immunohistochemistry; LDL-A, low-density lipoprotein apheresis; mes, mesangial deposits; MP, methylprednisolone; ND, not described; PSL, prednisolone; sCr, serum creatinine

The predominant subclass of antibodies to PLA2R is IgG4 and predominant deposition of IgG4 in the GBM is a well-known feature of PLA2R-associated primary MN. Although IgG4 anti-PLA2R antibodies have not been proven to be responsible for MN, recent studies have suggested the possible mechanisms of podocyte injury mediated by anti-PLA2R antibodies [24, 25]. IgG4-producing plasma cells by IgG4-RD may promote PLA2R antibody production, thereby contributing to the pathogenesis of PLA2R-associated MN.

In situations where PLA2R-positive MN and IgG4-RD coexist, given the possible involvement of IgG4-RD in the pathogenesis of PLA2R-associated primary MN, it is particularly difficult to completely discern whether MN is “primary” MN without any contribution form IgG4-RD or PLA2R-positive “secondary” MN contributed by IgG4-RD. When the responsiveness to steroid treatment suggests the contribution of IgG4-RD to the pathology of MN, as in this case, it may be necessary to perform maintenance therapy for assuming the contribution of IgG4-RD, even if PLA2R is positive.

In summary, the present case of PLA2R-positive MN preceding the onset of IgG4-RD highlighted the difficulty of distinguishing between primary and secondary MN in the presence of IgG4-RD. Although further studies are needed to clarify the relationship between PLA2R-positive MN and IgG4-RD, it may be worth keeping in mind that predisposition to IgG4-RD may be hidden in the background of a diagnosis of PLA2R-positive primary MN.
